# Application of whole-cell biosensors for analysis and improvement of L- and D-lactic acid fermentation by *Lactobacillus* spp. from the waste of glucose syrup production

**DOI:** 10.1186/s12934-023-02233-9

**Published:** 2023-10-30

**Authors:** Ernesta Augustiniene, Ilona Jonuskiene, Jurgita Kailiuviene, Edita Mazoniene, Kestutis Baltakys, Naglis Malys

**Affiliations:** 1 Bioprocess Research Centre, Faculty of Chemical Technology, Kaunas University of Technology, Radvilėnų pl. 19, Kaunas, LT-50254 Lithuania; 2 Department of Silicate Technology, Faculty of Chemical Technology, Kaunas University of Technology, Radvilėnų pl. 19, Kaunas, LT-50270 Lithuania; 3 Roquette Amilina, J. Janonio g. 12, Panevėžys, LT-35101 Lithuania; 4 Department of Organic Chemistry, Faculty of Chemical Technology, Kaunas University of Technology, Radvilėnų pl. 19, Kaunas, LT-50254 Lithuania

**Keywords:** L-lactic acid, D-lactic acid, Lactic acid bacteria, Fermentation, Transcription factor, Whole-cell biosensors

## Abstract

**Background:**

Lactic acid is one of the most important organic acids, with various applications in the food, beverage, pharmaceutical, cosmetic, and chemical industries. Optically pure forms of L- and D-lactic acid produced via microbial fermentation play an important role in the synthesis of biodegradable polylactic acid. Alternative substrates, including by-products and residues from the agro-food industry, provide a cost-effective solution for lactic acid production and are a promising avenue for the circular economy.

**Results:**

In this study, the transcription factor (TF)-based whole-cell biosensor strategy was developed for the L- and D-lactic acid determination. It was cross validated with commonly used high-performance liquid chromatography and enzymatic methods. The utility of biosensors as an efficient analytical tool was demonstrated by their application for the lactic acid determination and fermentation improvement. We explored the ability of *Lacticaseibacillus paracasei* subsp. *paracasei, Lactobacillus delbrueckii* subsp. *lactis*, and *Lactobacillus amylovorus* to biosynthesize optically pure L-lactic acid, D-lactic acid or mixture of both from organic-rich residual fraction (ORRF), a waste of glucose syrup production from wheat starch. The fermentation of this complex industrial waste allowed the production of lactic acid without additional pretreatment obtaining yields from 0.5 to 0.9 Cmol/Cmol glucose.

**Conclusions:**

This study highlights the utility of whole cell biosensors for the determination of L- and D-forms of lactic acid. The fermentation of L-lactic acid, D-lactic acid and mixture of both by *L*. *paracasei, L*. *lactis*, and *L. amylovorus*, respectively, was demonstrated using waste of glucose syrup production, the ORRF.

**Supplementary Information:**

The online version contains supplementary material available at 10.1186/s12934-023-02233-9.

## Introduction

In recent years, increasing interest has been dedicated to biorefining systems as a more promising and sustainable production of fuels, materials, and chemicals. Biorefining aims to exploit the full value of the feedstock by consistently extracting and valorizing its components. Usually, biorefinery feedstock includes lignocellulose, crops, microbial biomass, residues from agriculture and forestry industries, and recycled biobased products [1].

In the agro-food industry, wheat grains are processed to produce bioproducts including native and modified starches, glucose syrups, proteins, ethanol, etc. [2]. Glucose syrup is a plant-based sugar, an alternative to conventional granulated sugar in the food and beverage industries [3]. As other industries, wheat biorefining face a challenge to meet a zero-waste target. Despite the emerging biorefining technologies are making important progress, the utilization of low-value feedstock remains problematic [4].

One of the organic acids efficiently produced via fermentation is a lactic acid. The global market size for this acid was valued at nearly USD 3 billion in 2021 and is expected to approach USD 6 billion by 2030 [5]. Lactic acid is used in pharmaceutical, food, and cosmetic industries and is an important precursor in the synthesis of different chemicals (acrylic, pyruvic, propionic acids, esters of lactic and acrylic acids, polymers, etc.) [6, 7]. Fermentation of optically pure L- and D-lactic acids allows the production of biodegradable polymers (polylactic acid (PLA)) with highly physical properties [8]. Lactic acid bacteria (LAB) are the leading producer of lactic acid due to their several advantages including ability to achieve high yields close to 1 g/g with a traditional carbon sources and with alternative substrates such as lignocellulose waste [9, 10] and agro-food industrial residue [11, 12]. There is a large selection of these bacterial strains, so no additional genetic modification is required to produce optically pure L- or D-lactic acids. Ultimately, LAB has the reputation of being ‘generally recognized as safe’ (GRAS) when the use of genetically modified microorganisms in food industries is severely regulated in the EU.

Recently, we have reported a quantitative evaluation of L- and D-lactate-inducible gene expression systems from *Escherichia coli*, *Cupriavidus necator* and *Pseudomonas* species [13]. Such an inducible system is composed of a transcription factor (TF) that activates transcription when is bound with the ligand and a cognate inducible promoter. In combination with a reporter gene, it can be applied as an analytical tool for the determination of extracellular metabolite concentration by monitoring the expression of the reporter protein as a measurable output [14–17].

**Fig. 1:**
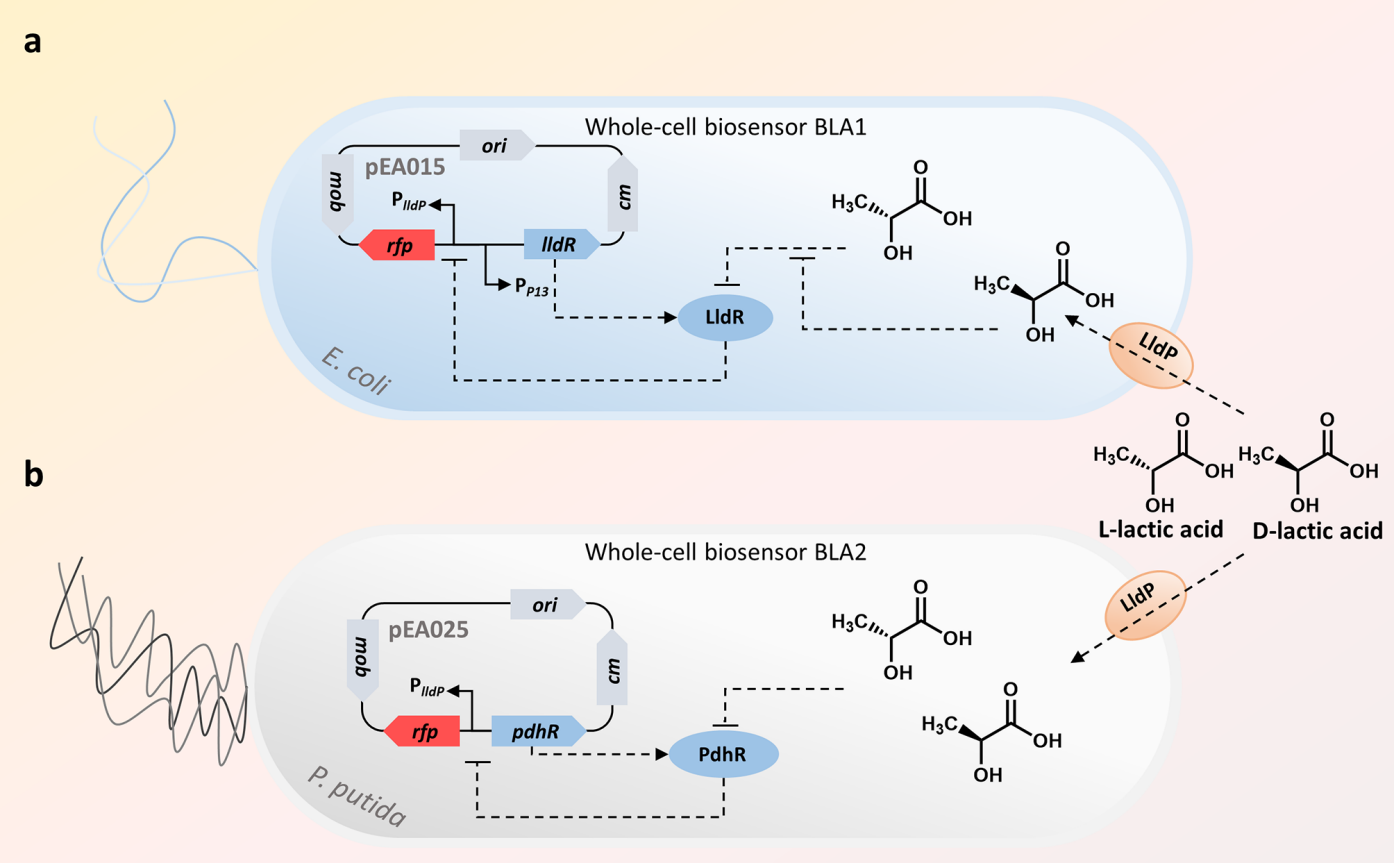
Whole-cell biosensors BLA1 and BLA2 for detection of L- and D-lactic acid. (a, b) Schematic of BLA1 and BLA2. The lactate permease LldP enables the transport of lactic acid into cells. *Ec*LldR and *Pf*PdhR belong to the GntR family of TRs acts as repressors in the absence of lactic acid [21–24]. The D-lactic acid inhibition of the interaction between L-lactic acid and LldR is shown. Plasmids pEA015 and pEA025 forming genes and essential promoters are indicated

In this study, the *E. coli* and *P. putida*-based whole-cell biosensors BLA1 (for L-lactic acid) and BLA2 (for LD-lactic acid) (Fig. 1) carrying previously characterized L- and D-lactate-inducible systems *Ec*LldR/P_*lldP*_ (plasmid pEA015) and BLA2 *Pf*PdhR/P_*lldP*_ (plasmid pEA025) [13] were validated as an efficient analytical tool for the L- and D-lactic acid determination. They were subsequently used to determine the concentrations of L- and D-lactic acid in biological samples. To produce either L-lactic acid, D-lactic acid or mixture of both, lactic acid bacteria *L. paracasei* [18], *L. lactis* [19], or *L. amylovorus* [20], respectively, were employed. The ability of *L. paracasei, L. lactis*, and *L. amylovorus* to produce the optically pure forms of lactic acid or their mixture from organic-rich residual fraction (ORRF), a waste of glucose syrup production, was examined by using BLA1 and BLA2. The key factors limiting the cell growth and the production of lactic acid were identified revealing strategies for the fermentation of lactic acid without chemical or enzymatic pretreatments of ORRF.

## Materials and methods

### ORRF and composition analysis

The ORRF was obtained as a waste byproduct remaining in the retentate after the crude glucose syrup ultrafiltration using undisclosed proprietary method (Roquette Amilina, Lithuania).

The biochemical compositions analysis of the ORRF was performed at Roquette Amilina. For carbohydrate analysis in ORRF a Dionex Ultimate 3000-4 HPLC system equipped with a refractive index detector (Thermo Fisher Scientific, USA) was used and chromatographic separation was obtained with an integrated Aminex HPX-87 H column (300 × 7.8 mm) (Bio-Rad, USA). The mobile phase was aqueous 5 mM sulfuric acid with a flow rate of 0.4 ml/min, a temperature of 60 °C, and an injection volume of 0.02 mL. Before analysis, carbohydrates in the ORRF sample were hydrolyzed using 60% sulfuric acid.

The lipids analysis in ORRF was carried out using the Thermo Ultimate 3000-3 HPLC system equipped with an ESA Corona Ultra CAD detector (Thermo Scientific™ Dionex™, USA) and the chromatographic separation was achieved using an integrated Fortis C8 column (1,7 μm; 50 × 2,1 mm) (Fortis Technologies, UK), thermostated at 40 °C. The mobile phase system consisted of solvent A (methanol: water: acetic acid 75:25:4 v/v/v) and solvent B (acetonitrile: methanol: tetrahydrofuran: acetic acid 50:37.5:12.5:4 v/v/v/v). Separation was carried out with a flow rate of 0.3 ml/min, and 3 µL of the lipid extraction suspended in chloroform: methanol (1:1 v/v) was injected into the column. The following system gradient was used to separate the lipid classes: 100% solvent A at 0 min, then solvent A decreased to 65% and solvent B increased to 45% over 3 min; then solvent A decreased to 32% and solvent B increased to 68% over 4 min; then solvent A decreased to 20% and solvent B increased to 80% over 3 min; then solvent A increased to 100% for 8 min; then solvent B increased to 100% for 8 min.

The protein amount of the ORRF sample was determined using the Dumas method with Thermo Flash 2000 instrument (Thermo Scientific, USA) with a factor of 6.25.

### LAB strains and fermentation conditions

Homofermentative LAB *Lacticaseibacillus paracasei* subsp. *paracasei* DSM 20,312 (*L. paracasei*), *Lactobacillus amylovorus* DSM 20,532 (*L. amylovorus*), and *Lactobacillus delbrueckii* subsp. *lactis* DSM 20,072 (*L. lactis*) were purchased from DSMZ (Germany). Routinely, LAB strains were grown in MRS medium containing 20 g/L glucose, 10 g/L enzymatic digest of casein, 5 g/L yeast extract, 10 g/L meat extract, 2 g/L K_2_HPO_4_, 5 g/L sodium acetate, 2 g/L diammonium hydrogen citrate, 0.2 g/L MgSO_4_, 0.05 g/L MnSO_4_, and 1.08 g/L Tween 80 (VWR Chemicals, USA) and used as inoculum.

For lactic acid fermentation, the ORRF was solubilized in distilled water at an initial concentration of 250 g/L and incubated for 1 h at 37 °C 200 rpm. After incubation, the mixture was autoclaved at 125 °C for 15 min at 1.5 atm pressure, then mixture was cooled and centrifuged for 5 min at 11,000 rpm. Water-insoluble substances including lipids were removed and the supernatants were collected. The resulting ORRF solution was diluted to 200 g/L, which contained 41.6 g/L (230.9 mM) of glucose monomer. In all cultivation cases, the ORRF solution contained 2 g/L K_2_HPO_4_, 0.2 g/L MgSO_4_, and 0.05 g/L MnSO_4_, and thereafter is referred to as ORRF minimal medium (ORRF-MM).

To evaluate the nutritional requirements of LAB strains for lactic acid production, the ORRF-MM was used as the sole nutrient source or with additional supplements including either 10% of MRS medium (v/v); 4 g/L of yeast extract (Sigma-Aldrich, USA); 8 g/L of meat extract (Fluka analytical, USA); 1% of vitamin supplement solution (v/v) that contained 2 mg/L folic acid, 10 mg/L pyridoxine hydrochloride, 5 mg/L riboflavin, 2 mg/L biotin, 5 mg/L thiamine, 5 mg/L nicotinic acid, 5 mg/L calcium pantothenate, 0.1 mg/L vitamin B12, 5 mg/L 4-aminobenzoic acid, 5 mg/L thioctic acid, and 900 mg/L monopotassium phosphate (ATCC, USA); 0.1% of Tween 80 (AppliChem GmbH, Germany) or their combination. To determine the optimal amount of nitrogen source, the ORRF-MM was supplemented with different concentrations of yeast extracts (1, 5, 10, 15, 20, and 30 g/L).

*L. paracasei* was grown at 30 °C, while *L. amylovorus* and *L. lactis* were grown at 37 °C in either MRS broth or ORRF-MM with supplements indicated above. Lactic acid production by LAB was evaluated in batch fermentation mode without shaking in 20 ml glass culture tubes containing 10 ml of media and under microaerophilic conditions using gas pack system Anaerocult (Merck, Darmstadt, Germany). The pH of media was manually maintained above 6 by addition of 10% KOH solution (w/w). Samples were aseptically withdrawn, and the growth curves were constructed by measuring the optical density at 600 nm (OD_600_) of the bacteria cultures using a spectrophotometer (BioMate 160 UV-Vis spectrophotometer, Thermo Scientific, USA). The glucose consumption and lactic acid concentration were determined as described below.

### HPLC and enzyme-based analyses

To perform the quantification of lactic acid and glucose, 1 ml sample was collected after 72 h of fermentation and centrifuged for 5 min at 15,000 rpm. The supernatant was saved and stored at -80 °C. Before analysis, the sample was filtered by passage through a 0.2 μm nylon filter (UptiDisc, Interchim).

HPLC analysis was performed with an Ultimate 3000 HPLC system equipped with a photodiode array (UV-VIS) detector (Thermo Fisher Scientific, USA) and an additional connected RefractoMax 521 refractive index detector (Thermo Fisher Scientific, USA). The chromatographic separation was obtained using a Rezex™ ROA-organic acid H+ (8%) (150 × 7.8 mm) (Phenomenex, Germany) equipped with a security guard column (Phenomenex Security Guard Cartridge (part number KJ0-4282)), thermostated at 25 °C. The mobile phase was aqueous 2.5 mM sulfuric acid with flow rate of 0.5 ml/min. The injection volume was 20 µl and samples were run for 35 min. Lactic acid and glucose were detected at 210 nm and identified according to retention times by comparing with the standards. Chromatograms were analyzed using Chromeleon 7 software (Thermo Fisher Scientific, USA).

For the determination of L- and D-lactic acid enantiomers, an enzymatic D-/L-Lactic acid (D-/L-lactate) (Rapid) assay kit (Megazyme, Ireland) was used and quantification of each lactate enantiomer was carried out according to the manufacturer’s recommendations.

### L- and D-lactic acid determination with whole-cell biosensors

The absorbance and fluorescence measurements of fermentation samples inoculated with biosensors was performed using Infinite M200 PRO (Tecan, Austria) microplate reader and a 96-well plate (Thermo Scientific, USA). Fermentation samples were diluted 5, 10, 15, 20 or 30 times depending on the expected concentration of lactic acid. The 7.5 µL of the fermentation sample or standard solution of L- or D- lactic acid were added to the 142.5 µL of exponentially growing BLA1 or BLA2 in M9 minimal medium supplemented with 1 µg/mL thiamine, 0.4 mM leucine, and 0.4% (w/v) glucose [25] at the absorbance A_600_ of 0.1 to 0.2. The RFP fluorescence and absorbance were quantified over time.

Standard aqueous solutions of sodium L-lactate (Alfa Aesar, Thermo Fisher Scientific, CAS 867-56-1) and sodium D-lactate (Sigma-Aldrich, CAS 920-49-0) were used to construct the dose-response curves.

#### L- or D-lactic acid determination in biological sample with one of lactic acid enantiomer

The concentration of L-lactic acid produced by *L. paracasei* was determined with BLA1, while the D-lactic acid produced by *L. lactis* was determined with BLA2. Absolute normalized fluorescence (*ANF*) was calculated as described previously [26]. For L- or D-lactic acid dose-response curves, values were used to plot the Hill function.

#### L- and D-lactic acid determination in biological sample with a mixture of lactic acid enantiomers

The *L. amylovorus* samples were analyzed with both biosensors BLA1 (for L-lactic acid concentration determination) and BLA2 (for total DL-lactic acid concentration determination) with additional recalculations described below. Total DL-lactic acid concentration was determined using the BLA2. BLA2 was tested with different concentrations of L- and D-lactic acid standards in the range of 0 to 20 mM over time and the *ANF* values for different L-lactic acid (*ANF*_*L*_) and D-lactic acid (*ANF*_*D*_) concentrations were calculated. *ANF*_*DL*_ for the total concentration of DL-lactic acid were calculated using formula (1) and the attended *k* coefficient, calculated according to formula (2).

1 $${ANF}_{DL}=\frac{{ANF}_{L}+{ANF}_{D}}{k}$$

2 $$k=\frac{{ANF}_{D}}{{ANF}_{L}}$$

For DL-lactic acid calibration curve, *ANF*_*DL*_ values were used to plot Hill function (3) [26]. Total DL-lactic acid concentration (*I*_*DL*_) in *L. amylovorus* fermentation supernatant samples was recalculated using the Hill function (3).

3 $${ANF}_{DL}\,or\, {ANF}_{L}\,or\, {ANF}_{D}={b}_{max}\times \frac{\left({I}_{DL}^{h}\, or\, {I}_{L}^{h}\, or\, {I}_{D}^{h}\right)}{{K}_{m}^{h}+{(I}_{DL}^{h}\, or\, {I}_{L}^{h}\, or\, {I}_{D}^{h})}+{b}_{min}$$

The parameters correspond to the maximum and minimum levels of RFP synthesis (*b*_*max*_ and *b*_*min*_, respectively), the concentration of total DL-, L- or D-lactic acid (${I}_{DL}$, ${I}_{L}$, or ${I}_{D}$, respectively), the Hill coefficient (*h*), and the inducer concentration, corresponding to the half-maximal reporter’s output (*K*_*m*_).

The concentration of L-lactic acid in *L. amylovorus* fermentation supernatant samples was determined using the BLA1. The concentration of D-lactic acid in *L. amylovorus* fermentation supernatant samples was calculated by subtracting the concentration of L-lactic acid (*I*_*L*_) from the total concentration of DL-lactic acid (*I*_*DL*_).

### Statistical analysis

All experiments were carried out using two or three biological-experimental replicates. The standard error of the mean was determined for each experimental sample time point. Linear regression analysis and unpaired *t*-test were performed using software GraphPad Prism 9. Bland–Altman analysis was performed using Microsoft Excel 2016.

## Results and discussion

### Application of biosensors for the optimization of L- and D-lactic acid fermentation

To differentiate L- and D-forms of lactic acid and increase the analysis throughput, the strategy of application of whole-cell biosensors was implemented in this study. Recently characterized inducible systems based on transcription factors *Ec*LldR and *Pf*PdhR [13] were used in biosensors BLA1 and BLA2 (Fig. 1a,b). The *Ec*LldR (BLA1) has been shown to be specific to L-lactic acid, whereas the *Pf*PdhR (BLA2) can be used for the detection of both enantiomers.

**Fig. 2:**
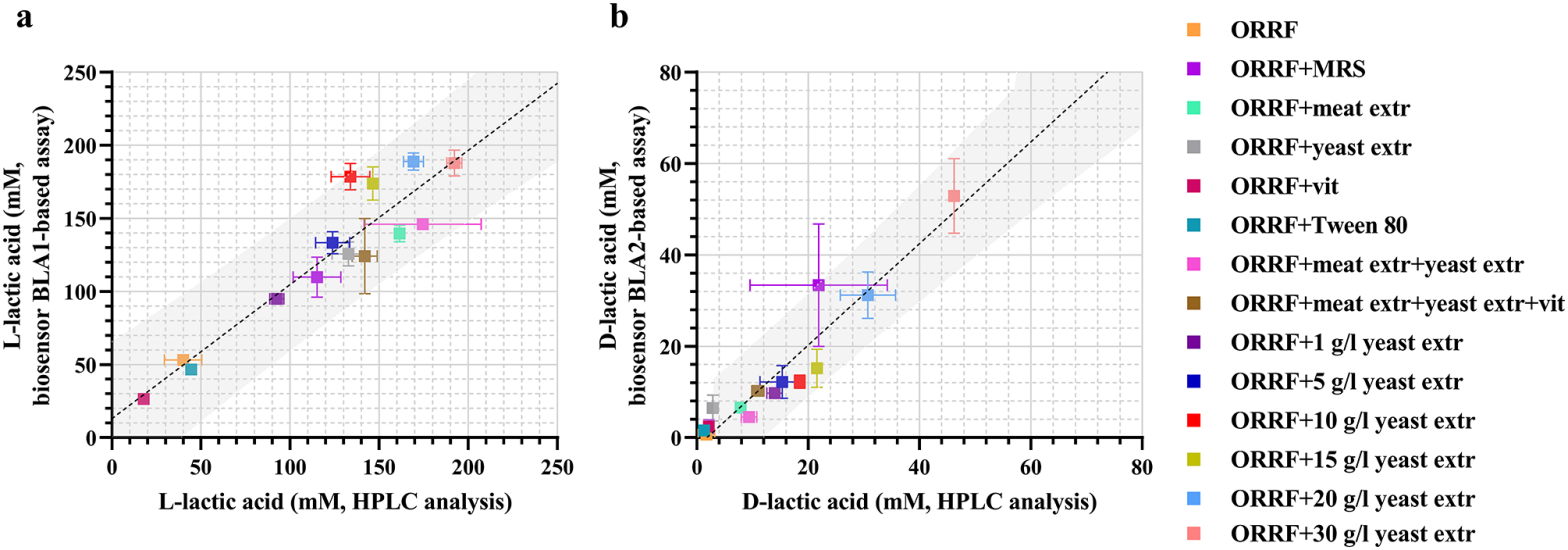
Linear regression (black dotted line) analysis of the correlation between the HPLC analytical method and the TF-based biosensors BLA1 (**a**) and BLA2 (**b**). Linear regression analysis was performed to find the 95% prediction interval (grey area). The concentrations of L- and D-lactic acid were obtained by assaying supernatant samples of *L. paracasei* and *L. lactis* fermentation collected at 72-hour. Error bars represent standard deviations of two biological replicates

#### Cross-validation of biosensors with HPLC-based analytical method

To evaluate biosensors’ suitability as an analytical tool, they were first cross-validated with the commonly used HPLC-based method. BLA1 and BLA2 were employed to investigate the L- or D-lactic acid fermentation by *L. paracasei* or *L. lactis*, respectively. The batch fermentation samples were collected at 72-hour as described in *Materials and methods* and the extracellular concentration of lactic acid was determined using biosensors or HPLC method. The whole-cell biosensors were logarithmically grown in MM and fluorescence output was quantified 6 h (BLA1) or 4 h (BLA2) after addition of fermentation sample. The lactic acid standards of concentration ranging from 0 to 20 mM were assayed simultaneously to construct dose-response curves (Supplementary Fig. S1 and Supplementary Table S1). These were used to estimate the concentration of lactic acid in the fermentation sample. The concentrations of L- and D-lactic acids in *L. paracasei* and *L. lactis* samples were compared with the results of the HPLC analysis (Fig. 2a,b). The coefficients *r* of correlation between concentrations estimated using biosensor assay and HPLC method were 0.84 and 0.89, indicating a fairly high accuracy of biosensor-assisted analysis. BLA1 and BLA2 enabled respectively to determine the L- and D-form of lactic acid, whereas the HPLC analysis did not allow discriminating between enantiomeric forms of lactic acid.

**Fig. 3:**
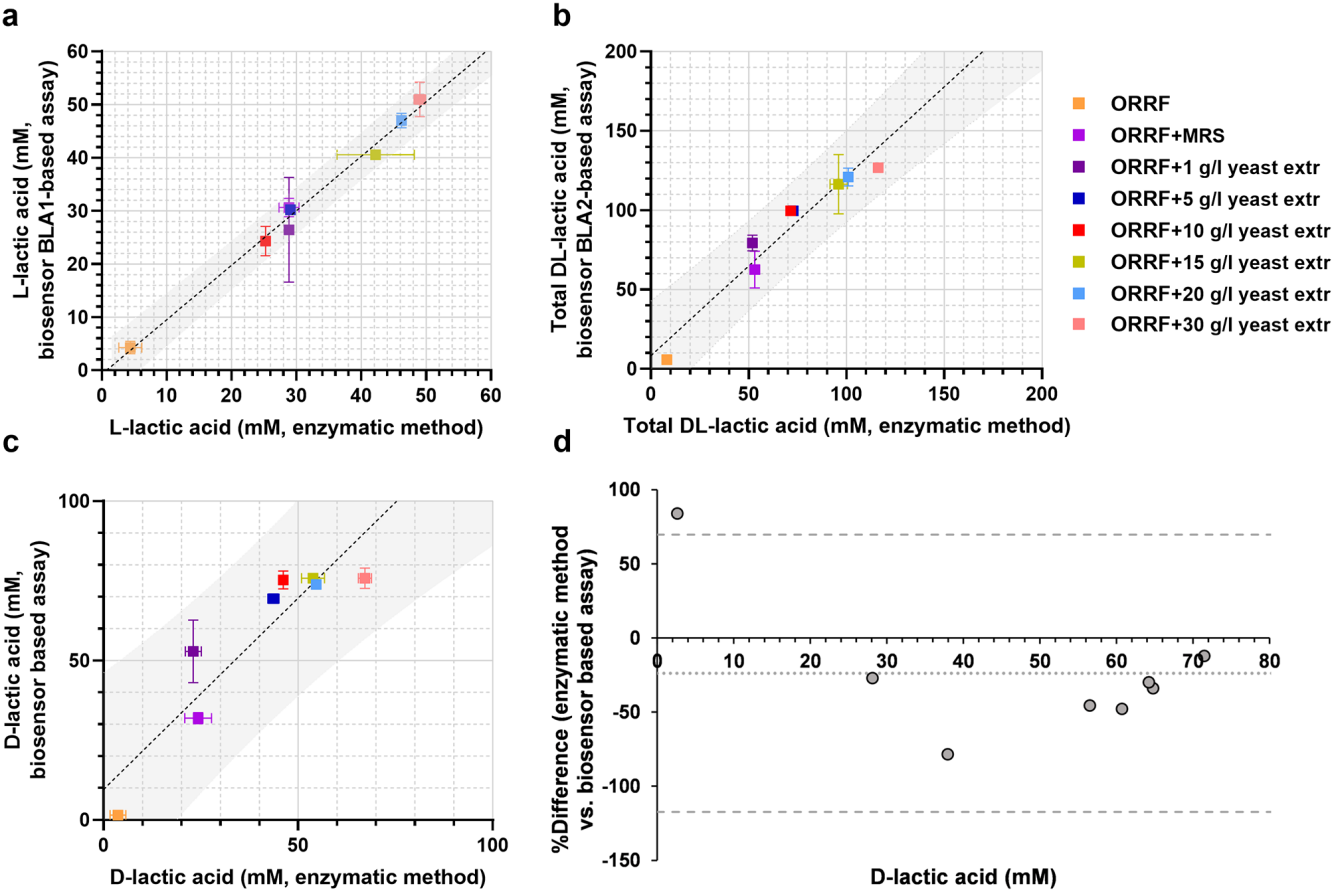
Comparison of assays with BLA1 and BLA2 to the enzymatic method for quantification of L- and D-lactic acid. (**a-c**) Linear regression analysis (black dotted line) of the correlation between the enzymatic method and the application of BLA1 and BLA2. Linear regression analysis was performed to find the 95% prediction interval (grey area). The concentrations L-lactic acid (**a**) and total DL-lactic acid (**b**) were obtained by assaying supernatant samples of *L. amylovorus* fermentation collected at 72-hour. The concentrations of D-lactic acid (**c**) were estimated as described in *Materials and methods* using data obtained by assaying supernatant samples of *L. amylovorus* fermentation. Error bars represent standard deviations of two biological replicates. (**d**) Bland Altman comparison plot (n = 8), showing the correlation between concentrations of D-lactic acid in *L. amylovoru*s fermentation samples, which were determined using biosensor-based assay and enzymatic method. The difference is plotted against average values, and the 95% limits of agreement (thick dashed lines) of the difference between the two methods of measurement are shown, as is the bias line (fine dashed lines)

#### Cross-validation of biosensors with enzyme-based analytical method

Separation and quantification of L- and D-lactic acids in racemic mixture requires specialized methods often based on use of chiral chromatography [27]. As an alternative, an expensive L-and D-lactate dehydrogenase enzyme-based assay can be used. In this study, we developed the whole-cell biosensor-based methodology for quantification of L- and D-lactic acid in the racemic mixture. To this end, *L. amylovorus* was used for the fermentation of the racemic mixture of lactic acid and the extracellularly excreted products were subjected to a 3-step analysis. First, the total concentration of DL-lactic acids was estimated using fluorescence output obtained with the BLA2 as described *Materials and Methods*. Second, in a similar way, the concentration of L-lactic acid in the racemic mixture was determined by applying the BLA1. Finally, the concentration of D-lactic acid was calculated by subtracting the concentration of L-lactic acid from the total concentration of D- and L-lactic acids.

It should be noted that a reduction of the fluorescence output by BLA1 was observed in the presence of D-lactic acid (Supplementary Fig. S2). Likely, the observed negative effect is due to a competitive inhibition by the D-lactic acid on the binding of L-lactic acid to the TR LldR (Fig. 1a). To circumvent the impact of inhibitory effect on the accuracy of estimation of L-lactic acid, the *L. amylovorus* fermentation sample was diluted to an approximately 7 mM of total lactic acid with an approximately 3 to 4 mM of D-lactic acid. This approach allowed to reduce the effect of D-lactic acid on the fluorescence output of BLA1 as shown in Supplementary Fig. S3.

To cross-validate biosensor-based method, the obtained titers of L- and D-lactic acid were compared to the concentrations determined using the enzymatic assay (Fig. 3a-c). The results showed a very good correlation between both types of assays with coefficient *r* of 0.95 for L-lactic acid (BLA1) and 0.98 for total lactic acid (BLA2). Notably, the correlation between the total concentration of lactic acid obtained with the BLA2 was slightly shifted towards higher concentrations (Fig. 3b). The estimated concentrations of D-lactic acid using the data obtained with both BLA1 and BLA2 showed a good correlation (*r* = 0.84) with enzymatic assay results (Fig. 3c). Similar to the total lactic acid, the shift toward higher concentrations was observed for D-lactic acid with the BLA2. To further evaluate the difference between the two groups of data, Bland–Altman comparison plot [28] was generated (Fig. 3d). It indicated the percentage difference of all the measurements obtained by the two analytical methods. D-lactic acid concentrations mean difference between the standard enzymatic method and the biosensors method was − 24% with a 95% limit of agreement ranging from 70 to 117%. In addition, Bland–Altman plot showed one of seven points (< 15%) was outside of the 95% limits of agreement, indicating the consistency between two methods.

The cross-validation results revealed that BLA1 and BLA2 can be used as analytical tools. They can be applied differential detection of L- and D-lactic acid in racemic mixture with a high accuracy, and at relatively low cost. Furthermore, the comparison to other analytical methods (Supplementary Table S2) revealed that the detection limit at the µmol level is similar to the HPLC [29] and enzymatic method. The application of whole-cell biosensor-based methods are significantly cheaper, approximately 30-fold, compared to the commercial enzymatic method [22], and do not require the complex equipment such as HPLC or LC-MS, and expensive chiral columns for the differentiation of lactic acid enantiomers [30].

### Investigation and improvement of lactic acid production from ORRF

The ORRF, obtained as an organic waste of glucose syrup production from wheat starch, was subjected to biochemical analysis (Supplementary Table S3). In addition to proteins and fatty acids, it revealed a high content of residual glucose, predominantly in a monomeric form (207.4 g/kg). Subsequently, ORRF was chosen as a potential substrate for the production of lactic acid. LAB *L. paracasei* [18], *L. lactis* [19], or *L. amylovorus* [20], known to produce either L-lactic acid, D-lactic acid or mixture of both, respectively, were used for fermentation.

**Fig. 4:**
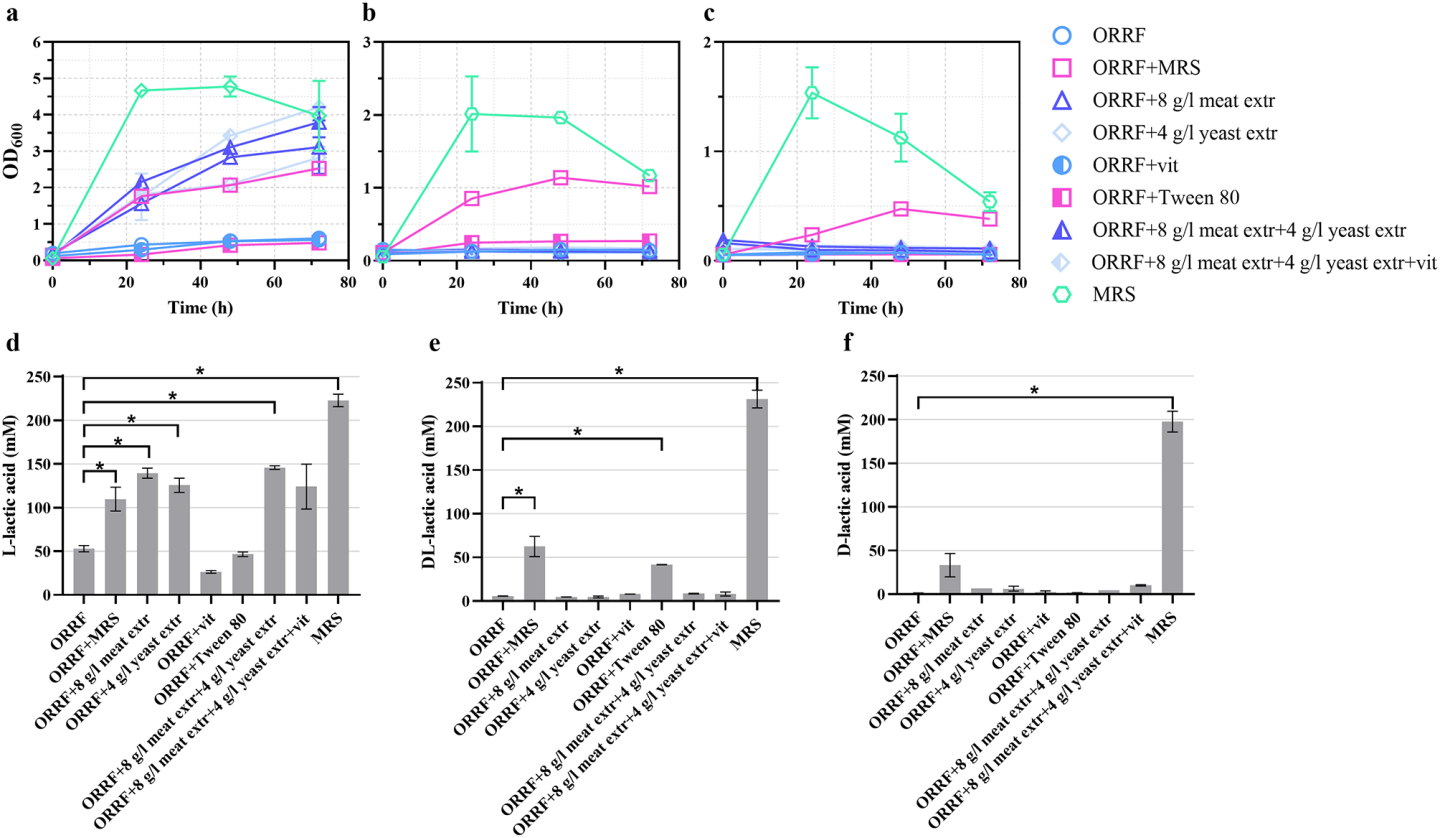
Growth and lactic acid concentrations obtained with (**a, d**) *L. paracasei*, (**b, e**) *L. amylovorus*, and (**c, f**) *L. lactis* in ORRF-MM with supplements. The medium contained 200 g/L ORRF including approximately 42 g/L (230.9 mM) of glucose. Lactic acid concentrations are determined at 72 h using BLA1 and BLA2. Substrates’ compositions are indicated. Error bars represent standard deviations of three biological replicates, *p < 0.05 (unpaired *t*-test)

Strains of the *Lactobacillus* were subjected to growth for 72 h using ORRF as a carbon source (Fig. 4a-c) and the titer of lactic acid was determined using biosensors BLA1 and BLA2 (Fig. 4d-f). When *L. paracasei*, *L. amylovorus*, and *L. lactis* were grown with ORRF as the sole nutrient source, the growth and lactic acid production were extremely low compared to the MRS medium. To identify nutrients required for growth, first, ORRF-MM was supplemented with 10% of MRS medium. The MRS supplementation improved the growth of *L. paracasei*, *L. amylovorus*, and *L. lactis* (Fig. 4a-c). It also contributed to the increase of lactic acid production from 53.0 ± 3.5 to 109.8 ± 13.6, from 5.8 ± 0.3 to 62.6 ± 11.6, and from 0 to 33.4 ± 13.4 mM, respectively (Fig. 4d-f), and the yield (Supplementary Fig. S4).

**Fig. 5:**
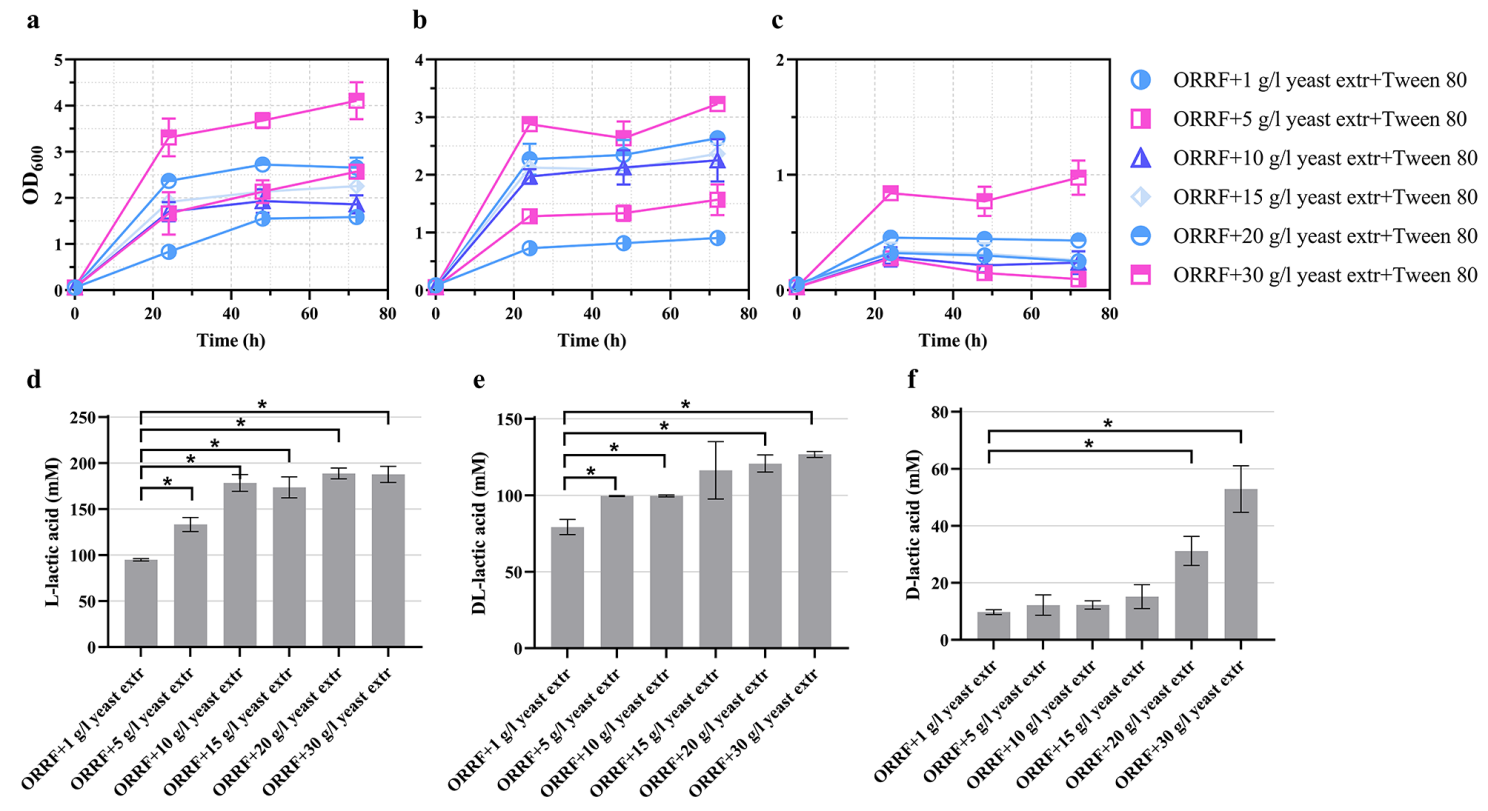
Growth and lactic acid concentrations obtained with (**a, d**) *L. paracasei*, (**b, e**) *L. amylovorus*, and (**c, f**) *L. lactis*. The medium contained 200 g/L ORRF including approximately 42 g/L (230.9 mM) of glucose. All cultures were supplemented with 0.1% of Tween 80 and yeast extract as indicated. Lactic acid concentrations are determined at 72 h using BLA1 and BLA2. Substrates’ compositions are indicated. Error bars represent standard deviations of three biological replicates, *p < 0.05 (unpaired *t*-test)

To determine the limiting factor for LAB growth, the ORRF-MM was supplemented with individual MRS broth components or their mixtures. The results showed that the L-lactic acid production by *L. paracasei* increased approximately 3-fold (up to 150 mM) when an additional nitrogen source (yeast or meat extract) was added to the ORRF-MM (Figs. 4). Supplementation with vitamins and Tween 80 had no significant effect on all three strains (Fig. 4). However, the addition of a nitrogen source (yeast extract) in combination with Tween 80 had a positive effect on *L. amylovorus* and *L. lactis* growth and lactic acid production (Fig. 5b, c, e and f). Notably, the positive influence of Tween 80 on *Lactobacillus* growth has been reported previously [31, 32].

To explore the optimal nitrogen source concentration for the *L. paracasei* strain, ORRF-MM was supplemented with different concentrations of yeast extract ranging from 1 to 30 g/L (Fig. 5a,d). Yeast extract was chosen as a less expensive alternative to meat extract to achieve a more economical process and to reduce the use of nitrogen substrates of animal origin for the commercial production of value-added chemicals [33]. The improvement in cell growth and titers of L-lactic acid was observed when higher concentration of yeast extract was present in the medium. The titer of L-lactic acid increased from 94.9 ± 1.3 to 187.7 ± 8.8 mM. Similar proportional increase of the LAB growth and lactic acid productivity, dependent on the yeast extract dosage, was observed previously [34]. It should be noted that the elevation of yeast extract concentration did not improve the yield of L-lactic acid (Supplementary Fig. S5). On the contrary, a decrease in the yield was observed indicating that the excess of nitrogen contributed to the production of biomass.

Similarly, *L. amylovorus* and *L. lactis* strains were grown in ORRF-MM supplemented with different concentrations of yeast extract ranging from 1 to 30 g/L (Fig. 5b, c, e and f) enabling to obtain titers from 79.3 ± 4.9 to 126.7 ± 2.0 mM of mixture of DL-lactic acids and from 9.8 ± 0.8 to 52.9 ± 8.2 of D-lactic acid, respectively (Fig. 5e,f). The estimated yields revealed comparable results to the *L. paracasei* strain (Supplementary Fig. S5).

For all three strains, the highest lactic acid yield was obtained when ORRF-MM medium was supplemented with 1 g/L of yeast extract. The addition of yeast extract eliminates the nitrogen deficiency in the medium and ensures the production of lactic acid.

## Conclusions

To create a circular economy as part of sustainable global production processes, it is important to transform industrial wastes such as agro-food industrial residues into value-added materials [35]. The search for new substrates is important not only for reducing environmental pollution but also for producing lactic acid more economically. This study demonstrated strategies for the fermentation of lactic acid using the waste of glucose syrup production and the process optimization using whole cell biosensors for lactic acid detection. The organic-rich residual fraction was used as a substrate for *Lactobacillus* strains without additional chemical or enzymatic pretreatment. *L. amylovorus* and *L. lactis* strains additionally required a nitrogen source and Tween 80. For the optimization of media formulation and improvement fermentation yields, the application of whole-cell biosensors BLA1 and BLA2 was shown as a reliable and high-throughput method for determining the concentration of lactic acid. The use of biosensors individually or in parallel enabled to generate data suitable for the determination of L- and D-form in fermentation samples containing pure or mixture of enantiomers. For further improvement of lactic acid yield, achieving an optimal carbon to nitrogen ratio is required [36]. Although the inducible system-reporter plasmids, forming foundation of BLA1 and BLA2, have not been adapted for other hosts, these biosensors can still be applied to the screening of lactic acid producers. This has been shown previously by co-cultivating both biosensor and producer cells [37] and it is demonstrated in this study determination of lactic acid concentration in the LAB culture supernatant. Ultimately, biosensors can be applied for the screening of L- or D-lactic acid producers, strain engineering, improvement of fermentation process, and contribute to the design–build–test–learn cycle in the broader context.

### Electronic supplementary material

Below is the link to the electronic supplementary material.

Supplementary Material 1
