# Correction: Application of whole-cell biosensors for analysis and improvement of L- and D-lactic acid fermentation by Lactobacillus spp. from the waste of glucose syrup production

**DOI:** 10.1186/s12934-023-02250-8

**Published:** 2023-12-07

**Authors:** Ernesta Augustiniene, Ilona Jonuskiene, Jurgita Kailiuviene, Edita Mazoniene, Kestutis Baltakys, Naglis Malys

**Affiliations:** 1 Bioprocess Research Centre, Faculty of Chemical Technology, Kaunas University of Technology, Radvilėnų pl. 19, Kaunas, LT-50254 Lithuania; 2 Department of Silicate Technology, Faculty of Chemical Technology, Kaunas University of Technology, Radvilėnų pl. 19, Kaunas, LT-50270 Lithuania; 3 Roquette Amilina, J. Janonio g. 12, Panevėžys, LT-35101 Lithuania; 4 Department of Organic Chemistry, Faculty of Chemical Technology, Kaunas University of Technology, Radvilėnų pl. 19, Kaunas, LT-50254 Lithuania

**Correction: Microbial Cell Factories (2023) 22:223**

10.1186/s12934-023-02233-9

In the original publication of the article, the figures 2 and 3 were swapped by mistake though the legends were correctly processed. The original article [1] has been corrected.

**Fig. 2:**
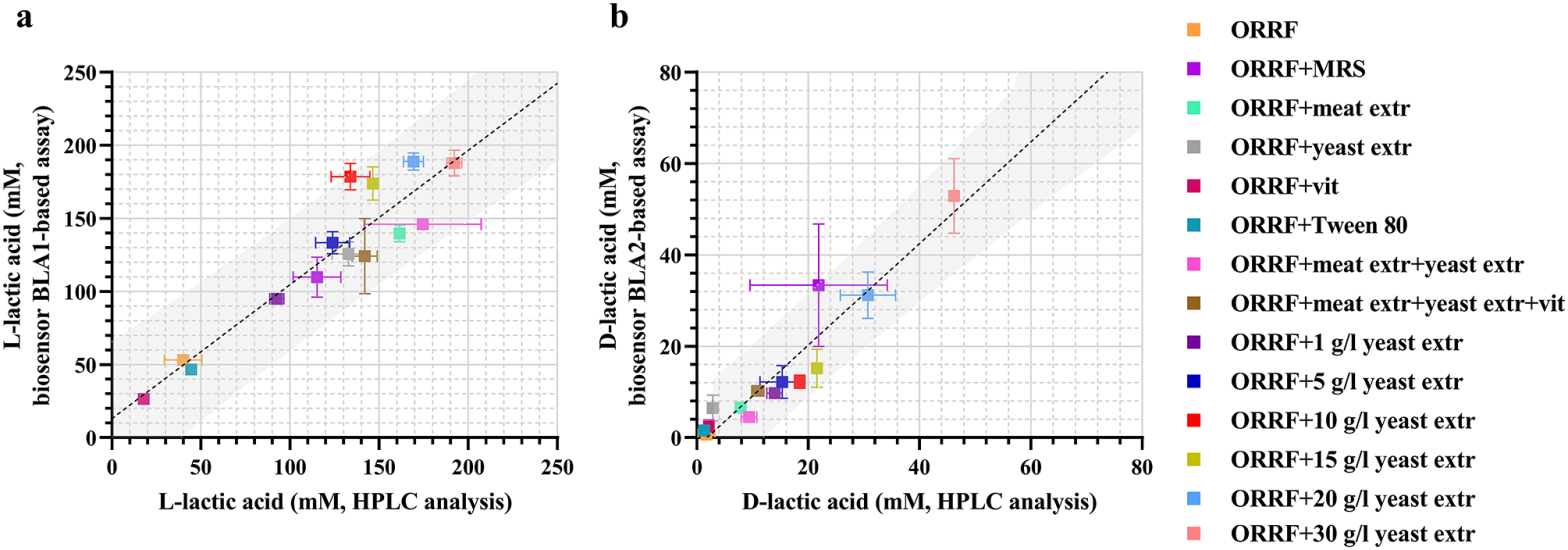
Linear regression (black dotted line) analysis of the correlation between the HPLC analytical method and the TF-based biosensors BLA1 (**a**) and BLA2 (**b**). Linear regression analysis was performed to find the 95% prediction interval (grey area). The concentrations of L- and D-lactic acid were obtained by assaying supernatant samples of *L. paracasei* and *L. lactis* fermentation collected at 72-hour. Error bars represent standard deviations of two biological replicates

**Fig. 3:**
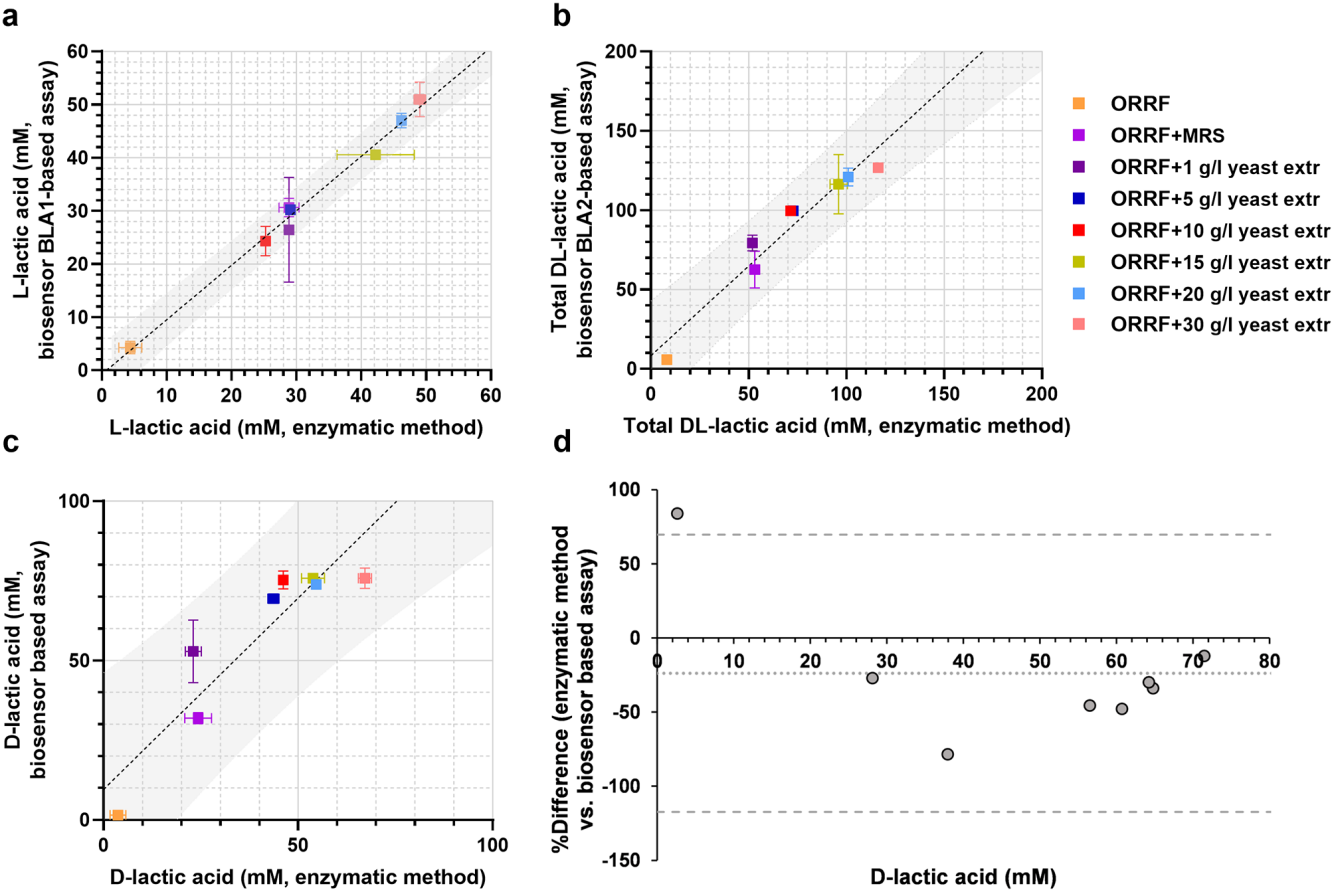
Comparison of assays with BLA1 and BLA2 to the enzymatic method for quantification of L- and D-lactic acid. (**a-c**) Linear regression analysis (black dotted line) of the correlation between the enzymatic method and the application of BLA1 and BLA2. Linear regression analysis was performed to find the 95% prediction interval (grey area). The concentrations L-lactic acid (**a**) and total DL-lactic acid (**b**) were obtained by assaying supernatant samples of *L. amylovorus* fermentation collected at 72-hour. The concentrations of D-lactic acid (**c**) were estimated as described in *Materials and methods* using data obtained by assaying supernatant samples of *L. amylovorus* fermentation. Error bars represent standard deviations of two biological replicates. (**d**) Bland Altman comparison plot (n = 8), showing the correlation between concentrations of D-lactic acid in *L. amylovoru*s fermentation samples, which were determined using biosensor-based assay and enzymatic method. The difference is plotted against average values, and the 95% limits of agreement (thick dashed lines) of the difference between the two methods of measurement are shown, as is the bias line (fine dashed lines)

## References

[1] Augustiniene, E., Jonuskiene, I., Kailiuviene, J. et al. Application of whole-cell biosensors for analysis and improvement of L- and D-lactic acid fermentation by Lactobacillus spp. from the waste of glucose syrup production. Microb Cell Fact 22, 223 (2023). 10.1186/s12934-023-02233-9 10.1186/s12934-023-02233-9 PMC10614324 37899432

