# Supplementary material for: Application of whole-cell biosensors for analysis and improvement of L- and D-lactic acid fermentation by Lactobacillus spp. from the waste of glucose syrup production
Source: Microb Cell Fact. 2023 Oct 30;22:223. doi: 10.1186/s12934-023-02233-9 (PMC10614324; doi:10.1186/s12934-023-02233-9)
Supplement: Supplementary file 1 — Supplementary Material 1 [file 12934_2023_2233_MOESM1_ESM.pdf]

Application of whole-cell biosensors for analysis and improvement of L- and D-lactic acid fermentation by *Lactobacillus* spp. from the waste of glucose syrup production

Ernesta Augustiniene[a,b], Ilona Jonuskiene[a], Jurgita Kailiuviene[c], Edita Mazoniene[c], Kestutis Baltakys[b] and Naglis Malys[a,d]*

[a]Bioprocess Research Centre, Faculty of Chemical Technology, Kaunas University of Technology, Radvilėnų pl. 19, LT-50254 Kaunas, Lithuania

[b]Department of Silicate Technology, Faculty of Chemical Technology, Kaunas University of Technology, Radvilėnų pl. 19, LT-50270 Kaunas, Lithuania

[c]Roquette Amilina, J. Janonio g. 12, LT-35101 Panevėžys, Lithuania

[d]Department of Organic Chemistry, Faculty of Chemical Technology, Kaunas University of Technology, Radvilėnų pl. 19, LT-50254 Kaunas, Lithuania

*Corresponding author, email: naglis.malys@ktu.lt

Supplementary Tables

Supplementary Table S1. Parameters of the dose-response curves of the BLA1 and BLA2 biosensors.

**Biosensor**	**Inducer**	B_{max}	B_{min}	K_m **(mM)**	**Hill coefficient**
BLA1 a	L-lactic acid	1845	79.52	0.2915	1.260
BLA1 b	L-lactic acid	1504	64.47	0.5461	1.348
BLA1 c	L-lactic acid	1220	63.32	0.4861	1.224
BLA2 a	D-lactic acid	304.6	14.85	1.796	0.8852
BLA2 b	D-lactic acid	269.0	14.83	1.700	1.047
BLA2 c	D-lactic acid	246.1	8.602	1.587	1.508

Supplementary Table S2. Advantages and disadvantages of analytical methods for the determination of lactic acid

Method	Analyte	Cost effectiveness	Time efficiency	Reliability	Throughput	Linear dynamic range, mM (limit of detection)	References
Enzymatic method	L-lactic acid and D-lactic acid	low	low	high	low	0.0056-0.333 (0.0023)	manufacturers manual[a]
HPLC	lactic acid	very low	low	very high	very low	0.025-5.0 (0.0061)	(Chen, Mowery, Castleberry, Walsum, & Chambliss, 2006)
LC-MS	lactic acid	very low	low	very high	very low	0.0038-0.379 (<0.0001)	(Ibáñez & Bauer, 2014)
Genetically encoded TF- and FRET-based biosensor	L-lactic acid	high	medium	high	very high	0.00076-0.0517 (0.00068)	(Xu et al., 2022)
TF-based biosensor	L-lactic acid	high	medium	high	very high	0.08-4.0 (0.001-0.05)	(Goers et al., 2017)
TF-based biosensor	L-lactic acid (BLA1 biosensor)	high	medium	high	very high	0.039-20 (0.0097)	*this work*
TF-based biosensor	D-lactic acid (BLA2 biosensor)	high	medium	high	very high	0.078-10 (0.0195)	*this work*

[a]D-/L-Lactic Acid (D-/L-Lactate) (Rapid) Assay Kit, Megazyme, Ireland

Supplementary Table S3. Biochemical compositions of organic-rich residual fraction (ORRF) remaining as a waste of glucose syrup production from wheat starch.

**Biochemical composition**	**ORRF**
Protein, amino acids, nucleic acids (g/kg)	***123.0***
Carbohydrates (g/kg) including:	***223.0***
Glucose (g/kg)	207.4
Xylose (g/kg)	8.2
Arabinose (g/kg)	1.6
Other carbohydrates (g/kg)	5.8
Lipids (g/kg) including:	***179.0***
Free fatty acids (g/kg)	114.0
Lysophospholipids (g/kg)	16.0
Phosphodiglycerides (g/kg)	36.0
Diglycerides (g/kg)	3.0
Triglycerides (g/kg)	7.0
Fatty alcohols (g/kg)	3.0
Organic acids (g/kg) including:	***27.6***
Lactate (g/kg)	1.0
Acetate (g/kg)	0.7
Other organic acids (g/kg)	25.9
Glycerol (g/kg)	***3.0***
Ash (%)	***44.4***

Supplementary Figures

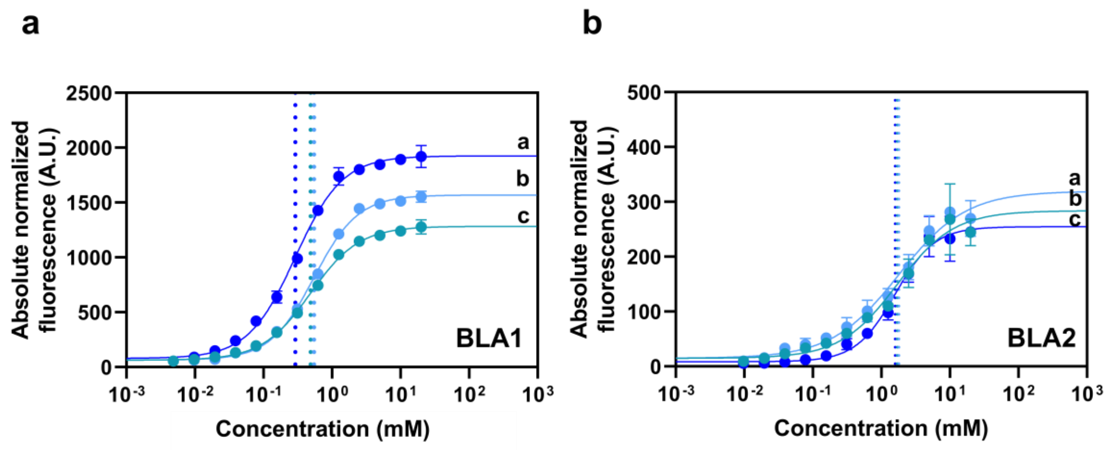

Supplementary Fig. S1. Dose–response curves of BLA1 and BLA2 biosensors. Absolute normalized fluorescence of (a) BLA1 6 h after the addition of different concentrations of L-lactic acid and absolute normalized fluorescence of (b) BLA2 4 h after the addition of different concentrations of D-lactic acid, ranging from 0 to 20 mM. Dose–response curves were fitted using the Hill function as described in *Materials and Methods* section. Error bars represent standard deviations of three biological replicates.

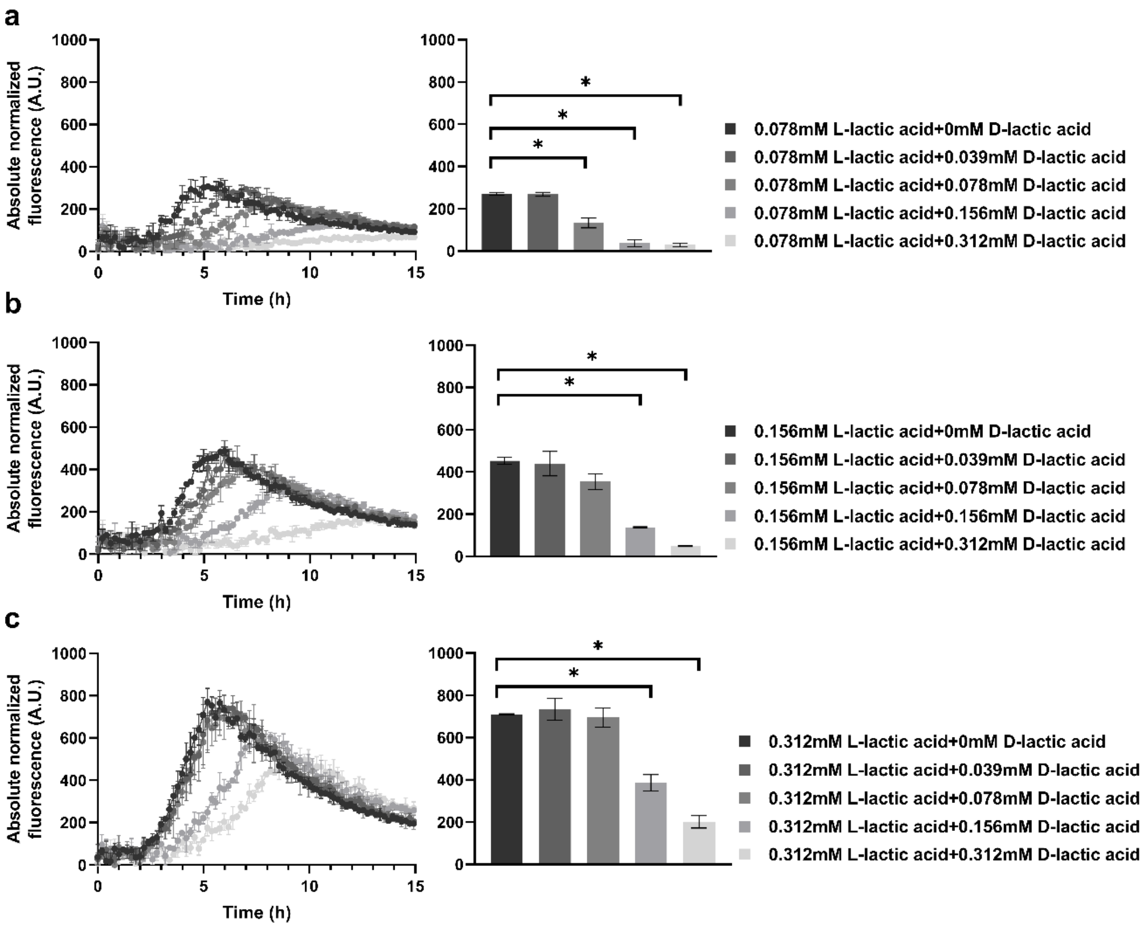

Supplementary Fig. S2. Absolute normalized fluorescence (on the left) of genetically encoded biosensor BLA1 in M9 minimal medium supplemented with mixtures of L-lactic acids and D-lactic acid. Single time-point fluorescence measurements (on the right) were taken 6 h after the addition of different mixtures of L-lactic acid and D-lactic acid. Error bars represent standard deviations of three biological replicates, *p < 0.001 (unpaired *t*-test).

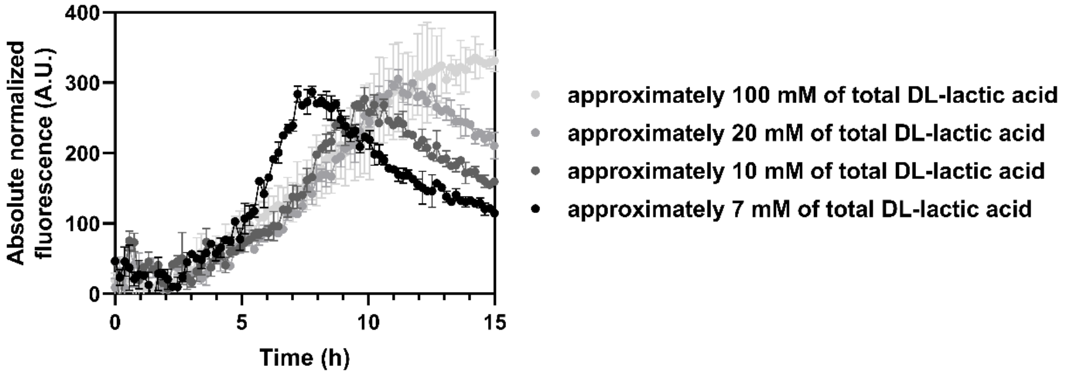

Supplementary Fig. S3. Absolute normalized fluorescence of genetically encoded biosensor BLA1 in M9 minimal medium supplemented with different concentrations of total DL-lactic acid of undiluted and with different dilutions *L. amylovorus* fermentation samples collected at 72 h. *L. amylovorus* was grown using 200 g/l of ORWF supplemented with 20 g/l of yeast extract, and 0.1% Tween 80. Error bars represent standard deviations of three biological replicates.

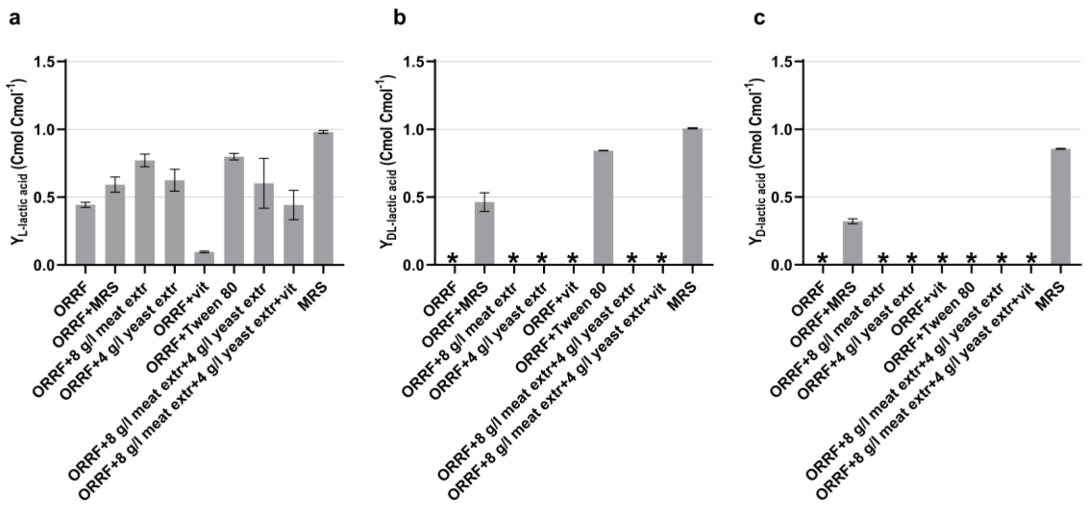

Supplementary Fig. S4. Lactic acid yields (Cmol Cmol^{-1} glucose) produced by (a) *L. paracasei*, (b) *L. amylovorus,* and (c) *L. lactis* after 72 h of fermentation. Nutrient supplements used in lactic acid production are indicated. *-no growth was observed and yields were not estimated. Error bars represent standard deviations of two biological replicates.

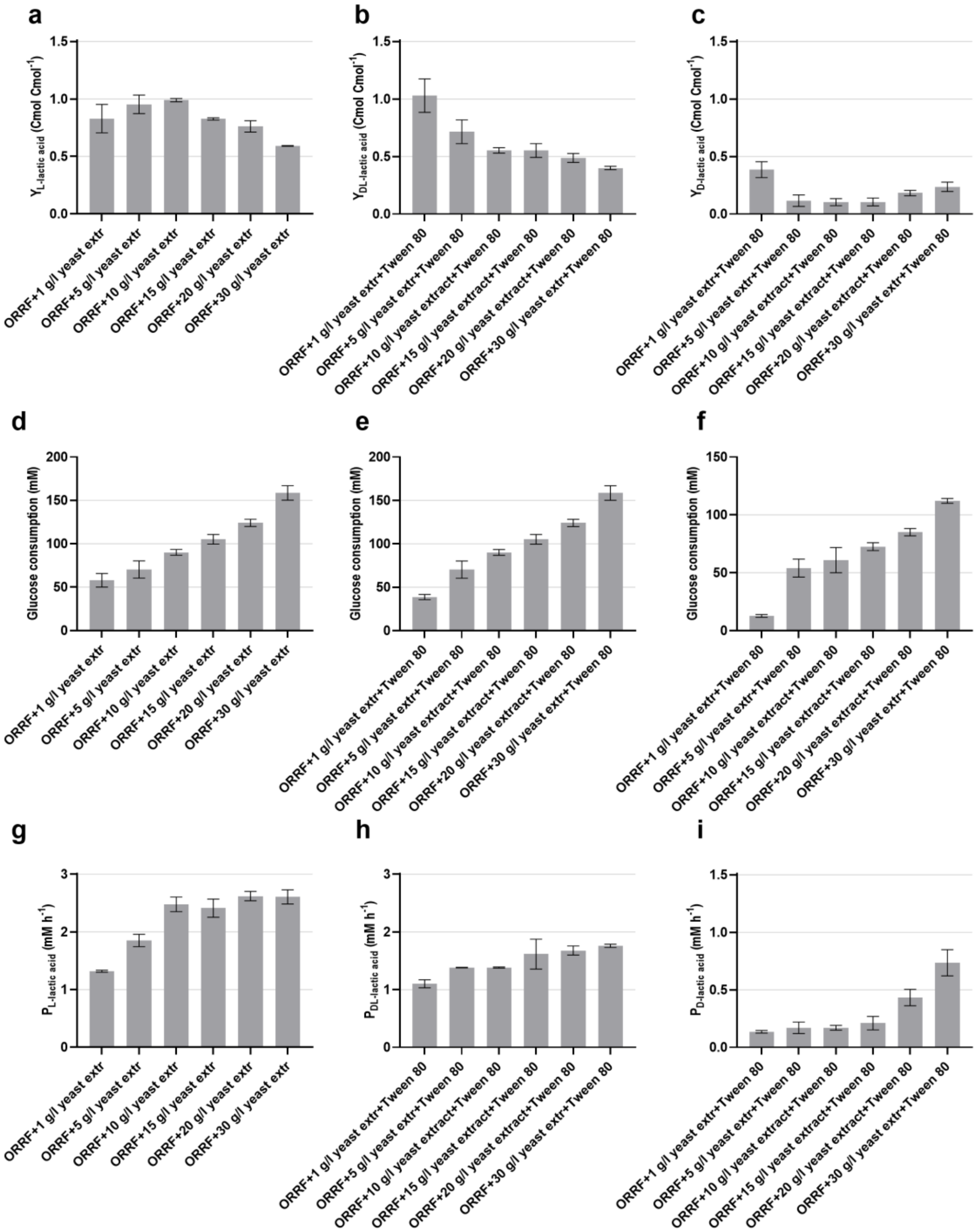

Supplementary Fig. S5. Lactic acid yields (Cmol Cmol^{-1} glucose), concentration of consumed glucose (mM), and fermentation productivity (mM h^{-1}) by (a, d, g) *L. paracasei*, (b, e, h) *L. amylovorus*, and (c, f, i) *L. lactis* after 72 h of fermentation. Nutrient supplements used in lactic acid production are indicated. Error bars represent standard deviations of two biological replicates.

References

Chen, S.-F., Mowery, R., Castleberry, V., Walsum, G., & Chambliss, C. (2006). High-performance liquid chromatography method for simultaneous determination of aliphatic acid, aromatic acid and neutral degradation products in biomass pretreatment hydrolysates. *Journal of Chromatography A*, *1104*(1-2), 54-61. https://doi.org/10.1016/j.chroma.2005.11.136.

Goers, L., Ainsworth, C., Goey, C. H., Kontoravdi, C., Freemont, P. S., & Polizzi, K. M. (2017). Whole-cell *Escherichia coli* lactate biosensor for monitoring mammalian cell cultures during biopharmaceutical production. *Biotechnology and Bioengineering, 114*(6), 1290-1300. https://doi.org/10.1002/bit.26254.

Ibáñez, A. B., & Bauer, S. (2014). Analytical method for the determination of organic acids in dilute acid pretreated biomass hydrolysate by liquid chromatography-time-of-flight mass spectrometry. *Biotechnology for Biofuels, 7*(1), 145. https://doi.org/10.1186/s13068-014-0145-3.

Xu, X., Xu, R., Hou, S., Kang, Z., Lü, C., Wang, Q., Zhang, W., Wang, X., Xu, P., Gao, C., & Ma, C. (2022). A selective fluorescent L-lactate biosensor based on an L-lactate-specific transcription regulator and Förster resonance energy transfer. *Biosensors, 12*(12), 1111. https://doi.org/10.3390/bios12121111.
